# Encouraging impulsive adolescents attending college to eat more fruit and vegetables: A preliminary investigation of negative urgency, message format and frame

**DOI:** 10.1177/13591053251375237

**Published:** 2025-10-07

**Authors:** Joanna Slodkowska-Barabasz, Susan Churchill, Nik Chmiel

**Affiliations:** 1 University of Chichester, UK; 2 University of Southampton, UK; 3 University of Sussex, Brighton, UK

**Keywords:** negative urgency, adolescents, fruit and vegetable consumption, narrative and non-narrative messages, loss- versus gain-framing

## Abstract

Adolescents high in negative urgency who are prone to emotion-driven impulsiveness and can be easily distracted, tend to eat unhealthily and may respond differently than those low in negative urgency to formatted and framed messages encouraging fruit and vegetables consumption. An experiment (*N* = 212) was conducted with a 2 (format: non-narrative vs narrative) × 2 (frame: loss vs gain) factorial design having participants’ level of negative urgency as a moderator. Findings revealed a three-way interaction between negative urgency, message format and frame. In the gain-framed condition, adolescents high on negative urgency were persuaded best by non-narrative messages, whereas those low on negative urgency were best persuaded by narrative messages. These findings provide initial evidence that recipients’ negative urgency influences how persuasive message framing and format are in encouraging adolescents to consume more fruit and vegetables. These results have implications for the construction of effective health appeals to adolescent populations.

## Introduction

Eating a healthy diet in adolescence is important for growth, long-term health, and the development of healthy eating practices (da Costa et al., 2024). Adolescent dietary behaviours can be influenced by a variety of factors, for example, individual (e.g. gender, ethnicity, BMI), social (e.g. parental and peer influence, culture), and environmental (e.g. unhealthy foods advertising, food affordability and accessibility; Heslin and McNulty, 2023; Müller et al., 2024) most of which cannot be changed by individually targeted health communications. Cognitions (i.e. nutritional knowledge and motivation to consider consequences of (un)healthy eating behaviours), however, are established modifiable factors in eating behaviours and can be changed with health promotion interventions (Raut et al., 2024). The evidence has established that people differ in their receptiveness to health information (Covey, 2014; Dudley et al., 2023) and in their predisposition to un/healthy eating (Allen et al., 2024), which warrants more research to provide a nuanced understanding of what message works for whom and in what context.

An important personality trait that has been shown to influence the healthiness of dietary habits is negative urgency (Coumans et al., 2018b; Smith et al., 2021). Negative urgency is a facet of impulsivity that reflects a tendency to act impulsively when experiencing negative emotions (Whiteside and Lynam, 2001). Adolescents typically experience elevated levels of negative urgency (Cyders and Smith, 2008; Littlefield et al., 2016) that are associated with insufficient impulse control and emotion regulation due to ongoing brain maturation processes (Defoe et al., 2015; Fisher-Fox et al., 2024a). Meta-analyses and longitudinal studies conducted in adolescent samples demonstrated predictive role of negative urgency in high-risk behaviours (Berg et al., 2015), including eating disorders (Davis et al., 2024) and poor diet quality (Smith et al., 2021), suggesting that those high in negative urgency behave in an impulsive way in order to alleviate negative affect (Becker et al., 2016). Recent research in adult and adolescent samples has shown that individuals high in negative urgency are more likely to eat insufficient amounts of fruit and vegetables (Churchill and Jessop, 2011; Potard, 2022) and tend to eat excessive amounts of foods that are high in fat, salt, and sugar (Booth et al., 2018; Coumans et al., 2018b). Such potential for low fruit and vegetable intake may put high negative urgency adolescents at increased risk of excessive weight gain and obesity (Adise et al., 2024; Coumans et al., 2018a) and negative health consequences later in life (Bo et al., 2014; Joshipura et al., 2001) making them a key target group for health communication intervention efforts designed to increase fruit and vegetable consumption.

Providing health information about the benefits of sufficient fruit and vegetable consumption can help to motivate dietary behaviour change (Vaitkeviciute et al., 2015; Wolfenden et al., 2021). The success of health messages, however, depends on whether a message can trigger in the reader the psychological processes that underlie persuasion – processing of health information (O’Keefe, 2015; Petty and Briñol, 2015). Broadly, health messages can evoke cognitive responses (e.g. critical or superficial thinking about the message) and affective responses (e.g. emotions such as fear, contentment, hope and anger) in the reader that in turn may lead to attitude and/or behaviour change (Kopfman et al., 1998; Nabi et al., 2020; Wagner and Petty, 2022). The extent of thinking invested in the analysis of the message arguments may be contingent on, for example, the relevance of the message to the readers’ situation (Evans, 2017) or on the enjoyment derived from effortful thinking (Braverman, 2008). Extensive research compiled evidence for multiple ways in which message-induced emotional reactions can act as an information-processing facilitator by prompting either low- or high-effort thinking and directing behavioural outcomes (Stavraki et al., 2021; Wagner and Petty, 2022). Yet, the effects of emotions in persuasion processes depend on the reader’s capacity to recognize and act upon emotions. Researchers noted that for persuasion to occur it is not sufficient just to elicit emotional responses in the reader, it is necessary for emotions to be perceived as relevant to the message topic (Dillard and Nabi, 2006). Reduced capacity to recognize emotions induced by the message may diminish the likelihood of understanding, evaluating and engaging with information in a message and may reduce chances for persuasion (Appel and Richter, 2010; Dillard and Nabi, 2006). It is possible that in adolescents high (vs low) in negative urgency having trouble managing negative emotions may not only be associated with their eating behaviour but it may also impact on how they process health information.

### Negative urgency and message processing

Certain characteristics of those with higher levels of negative urgency may influence how they respond to health messages. These relate to their handling of emotional content on the one hand and the effort they put into absorbing the message on the other. Research in adult populations suggests that those higher in negative urgency demonstrate a bias for immediate reward (Smith and Cyders, 2016), impaired emotion-related decision-making processes and a reduced ability to appraise and regulate emotions (Fisher-Fox et al., 2024b; Shishido et al., 2013). Individuals high (vs low) in negative urgency also demonstrate difficulties maintaining attention (Rosenthal et al., 2024; Sharma et al., 2013). In particular, studies with adolescent samples have shown that those reporting high levels of negative urgency tend to be less likely than those reporting low levels of negative urgency to consider the pros and cons of a given behaviour (Kulbida et al., 2024) and typically use low-effort (vs high-effort) processing when making health-related decisions (Canale et al., 2015). It is possible therefore that messages that do not rely on emotional content for their appeal, and that can be assimilated without much effort, are likely to be more successful in persuading high urgency adolescents of the benefits of eating more fruit and vegetables. In contrast, individuals low in negative urgency appear more open to subtle emotional appeals, and more willing to consider their pros and cons.

Despite the fact that those high and low in negative urgency potentially differ in their receptiveness to emotion-evoking and thought-provoking health messages, negative urgency has not been assessed in a persuasion context. In the current study, we chose two message construction strategies: formatting (narrative vs non-narrative) and framing (loss vs gain) that have been shown to generate different levels of cognitive and emotional reactions in readers (Hamby and Jones, 2022; Hinyard and Kreuter, 2007; Mays and Evans, 2017) to test their effectiveness in encouraging fruit and vegetable consumption for adolescents high and low in negative urgency. Below we discuss how narrative and non-narrative messages in a gain and a loss-frame may encourage different types of processing, suggesting that a non-narrative message in a gain-frame will likely be more persuasive for those high in negative urgency, whilst a narrative message in a gain-frame will be more likely to persuade those low in negative urgency to eat more fruit and vegetables.

### Message format: Non-narrative and narrative

Non-narrative messages, often seen as a traditional way to communicate health information, can include explicit health recommendations and lists of facts and statistics supporting them (Kreuter et al., 2007). The persuasive effects of non-narrative messages have been explained by dual-process models, where either low- or high-effort thinking about message arguments plays a key role (Kopfman et al., 1998; Petty and Cacioppo, 1986). Low-effort thinking involves a quick skim of the message, and more effortful thinking involves critical analysis of the arguments in a message. Both types of processing have been shown to promote changes in beliefs or behaviour (Evans, 2017; Griffin et al., 2002; Wagner and Petty, 2022).

In recent years, narrative-based strategies have been gaining ground as a useful health communication tool (Dudley et al., 2023; Hinyard and Kreuter, 2007; Oschatz and Marker, 2020). A positive health narrative, for example, uses a story-like format to recount the beneficial health outcomes experienced by a person following a change in lifestyle, such as giving up smoking, doing more exercise, increasing fruit and vegetable consumption, or reducing sugar intake (Gray and Harrington, 2011; Larkey and Gonzalez, 2007; Li, 2021; Kim et al., 2012). Narratives have a great potential to evoke emotions in the reader who sympathizes with the character (Hamby and Jones, 2022). In fact, readers are more likely to accept narrative message arguments when they become transported into the story, that is, when they understand and share emotions of the characters, pay attention to the story and create mental representations of the events depicted in the narrative (Green and Brock, 2002; Ratcliff and Sun, 2020).

The persuasive effects of narrative and non-narrative forms of communication on health outcomes are not uniform (Dudley et al., 2023; Zebregs et al., 2015) and tend to be a subject to contextual and/or individual characteristic factors (Xu, 2023). For example, research has demonstrated that an individual predisposition to engage with an emotional content can moderate the persuasiveness of narratives (vs non-narratives). Specifically, individuals with high levels of *need for affect* – a tendency to approach and seek out emotive stimuli, such as movies, stories, or events (Appel and Richter, 2010) and those high on a measure of *transportability* – who tend to become easily immersed into the narrative text (Mazzocco et al., 2010) are more likely to follow health recommendations depicted in a narrative as compared to a non-narrative message. It is possible then that individuals high in negative urgency who are not comfortable with their emotions and who are prone to distractions may not be drawn into the narrative message. A narrative message persuades best through effortful cognitive and emotional engagement with the story, processes that are more likely to be adopted by those low (vs high) in negative urgency. In contrast, a non-narrative message allows for persuasion through low-effort processing that does not rely on emotional appeal for its effect and so is likely to be more persuasive than other types of messages to those high in negative urgency.

### Message frame: Gain and loss

Prospect Theory (Kahneman and Tversky, 1979) proposes that the way in which presented information is framed as a gain or a loss can also impact the way messages are responded to. Health messages, therefore, can focus on the potential health benefits of acting healthily (i.e. a gain-framed appeal) or on the health costs of failing to act or acting unhealthily (a loss-framed appeal; Gallagher and Updegraff, 2012; Rothman and Salovey, 1997).

Gain-framed messages have been found to be processed without any strong accompanying emotional reaction, typically generating low levels of a positive emotional reaction (Nabi et al., 2020; Van’t Riet et al., 2010). On the other hand, loss-framed information tends to evoke stronger negative emotional reactions such as anxiety and nervousness (Mays and Evans, 2017; Zhao et al., 2023). We propose, therefore, that a gain-framed message will likely allow recipients differing in negative urgency to respond with their preferred or usual way of processing as outlined above. In contrast a loss-framed message will likely be accompanied by a relatively strong negative emotional reaction that could differentially influence how the content is processed for low and high negative urgency individuals.

Research has shown that loss-framed narratives tend to be less persuasive than gain-framed narratives (De Graaf et al., 2016) because they may elicit lower levels of transportation (Lee and Kim, 2022). We suggest this is consistent with the idea that a heightened negative emotional reaction is likely to reduce the range of emotional cues utilized by readers (c.f. Easterbrook, 1959), which may prevent readers of a narrative message from forming an emotional bond with the characters (Murphy et al., 2013). Notwithstanding the precise mechanisms involved we suggest that in the loss-frame, narrative messages will be compromised in their ability to transport readers, and this will affect those who are more open to persuasion through transportation, namely those low in negative urgency. Thus, the loss-frame will reduce the advantage in persuasion of narrative messages for those low in negative urgency.

Secondly, research has found that as well as evoking negative emotions in readers, loss-framed health information tended to persuade through effortful processing (Carfora et al., 2021; Leshner and Cheng, 2009). This implies that any appeal based on skimming a message may be interfered with or nullified. We suggest therefore that in the loss-frame, non-narrative messages will be compromised in their ability to persuade readers through skimming, and this will affect more those who prefer to process messages in this way, namely those high in negative urgency. Thus, the loss-frame will reduce the advantage in persuasion of non-narrative messages for those high in negative urgency.

Hence, for the first time we test whether individual differences in negative urgency moderate the effects of message format and frame on behaviour in the context of adolescent fruit and vegetable consumption. We expect:

H1: There will be a three-way interaction between negative urgency, message format, and message frame such that those high in negative urgency will be best persuaded by non-narrative messages in a gain-frame, whilst those low in negative urgency will be best persuaded by narrative messages in a gain-frame, and where these differences are minimized in the loss-frame.

## Method

### Design and procedure

The study employed a 2 (message format (non-narrative, narrative)) × 2 (message frame (loss, gain)) × negative urgency (continuous index)) longitudinal design, involving three waves of data collection by questionnaire, each separated by approximately 1 week. A power calculation revealed a minimum sample size of 141 was required in order to detect a medium effect size (*f*^2^) of 0.15 with a 0.80 level of power. Questionnaires were administered in classroom settings. Students who agreed to take part in the research completed a consent form and then the baseline questionnaire. Seven days later, participants as they were sat in class, were alternately allocated to one of the four experimental message conditions (format/frame) and asked to complete the corresponding time 1 questionnaire. The time 2 questionnaire was administered 7 days after that. At the end of the last testing session, participants were thanked and debriefed. Participants did not receive any compensation for their participation. This study was conducted following receipt of ethical approval by the Research Ethics Committee at the University of Chichester.

### Participants

Three hundred and thirty-two participants completed the baseline measures in classroom settings. Of these, 120 were absent when subsequent data was collected, representing an attrition rate of 28%, and leading to unequal numbers of participants in each condition as follows: non-narrative/loss (*n* = 44), non-narrative/gain (*n* = 43), narrative/loss (*n* = 68), narrative/gain (*n* = 57). Hence analyses were conducted on 212 older adolescents (73.4% female) who were aged between 16 and 20 years (*M* = 17.04; SD = 0.85). Given that age range 16 to 20 years old tends to be considered as older adolescence (e.g. Swamy et al., 2023; Zhang and Thompson, 2021) we refer to our sample as older adolescents. Participants’ body mass index (BMI) ranged from 15.20 to 38.20 (*M* = 22.16; SD = 3.95).

### Measures

#### Baseline questionnaire

##### Demographic information

Participants were asked to indicate their age, gender, weight and height. BMI was calculated for each participant: BMI = weight (kg)/(height [m])^2^.

##### Negative urgency

Individual differences in negative urgency were assessed using the 12-item Negative Urgency subscale from the UPPS Impulsive Behavior scale (Whiteside and Lynam, 2001). All items in this scale are behavioural-domain general, insofar as they assess impulsive tendencies in general rather than in the context of speciﬁc behaviours, for example, ‘When I am upset, I often act without thinking’. Responses to all items were given on a 4-point scale ranging from 1 (*agree strongly*) to 4 (*disagree strongly*). All items except one were reverse-scored. Higher scores reflect a greater tendency to act on impulse when faced with negative affect (Cronbach’s α = 0.85).

#### Time 1 questionnaire

##### Baseline fruit and vegetables consumption

Participants were asked, ‘Do you generally eat 5 portions of fruit and vegetables every day?’ Participants responded on a 5-point scale ranging from 1 (*never*) to 5 (*always*), with higher scores indicating higher levels of fruit and vegetable consumption.

##### Persuasive messages

Participants then read a health message in either a narrative or non-narrative format, which depicted either the health costs of insufficient fruit and vegetable consumption or the health benefits of sufficient intake of fruit and vegetables. The messages are provided in the Supplemental Material.

#### Time 2 questionnaire

##### Fruit and vegetable consumption

Following Thompson et al. (2000), frequency of fruit and vegetable consumption was assessed by eight items. Four items assessed the intake of fruits in the past week, that is, participants were asked to rate how often they had eaten fruits at different times of the day: breakfast, lunch, and dinner and between meals over the previous 7 days. Example item included: ‘Over the past 7 days, on how many days did you eat fruit for your breakfast?’ Four items assessed the intake of vegetables in the past week, that is, participants were asked to rate how often they had eaten vegetables at different times of the day: breakfast, lunch, and dinner and between meals over the previous 7 days (‘Over the past 7 days, on how many days did you eat vegetables for your dinner? Do not count potatoes’). Responses to items were given on an 8-point scale ranging from 0 (*none*) to 8 (*7* *days*). Responses were summed to provide a measure of fruit and vegetables consumption, with higher scores indicating greater levels of consumption.

### Statistical analyses

All data were analysed using SPSS (version 21; IBM, Chicago, IL, USA). We conducted *t*-tests for continuous variables and chi-squared tests for categorical variables to examine differences in sample characteristics and key study variables. Pearson correlations were calculated to determine bivariate associations between covariates and key study variables. Hierarchical multiple regression analysis was used to explore the impact of message format, message frame, and negative urgency on fruit and vegetable consumption controlling for past fruit and vegetable consumption, BMI and age. All continuous variables were standardized, and all categorical variables were dummy coded prior to analysis. Standardized values of negative urgency were used to create interaction terms that included negative urgency. At step 1, we controlled for age, as this variable had been found to differ across conditions, and for potential covariates of baseline frequency fruit and vegetable consumption, and BMI. At step 2, we entered message format (coded as: 0 = non-narrative, and 1 = narrative), message frame (coded as: 0 = loss, and 1 = gain), and negative urgency to explore whether there were any significant associations between these variables and fruit and vegetable consumption at time 2. The 3 two-way interaction terms (format × frame; format × negative urgency; frame × negative urgency) were entered at step 3, and the three-way interaction was entered at step 4. The dependent variable was fruit and vegetable consumption at time 2. Interactions were analyzed using the macro PROCESS for SPSS (Hayes, 2013). Following recommendations by Aiken and West (1991) for interpreting significant interaction effects, negative urgency was calculated in standard deviation (SD) units at high (1 SD above the mean) and low (1 SD below the mean) levels of negative urgency. The alpha level for statistical significance was set at *p* < 0.05.

## Results

### Preliminary analyses

One-way ANOVAs and chi-square tests revealed no significant differences between those who completed all three parts of the study and those who failed to respond beyond baseline in terms of BMI, negative urgency and gender (*p*s > 0.06). A significant difference was found between responders and non-responders with regards to baseline frequency of fruit and vegetable consumption and age, such that those who completed three parts of the study were slightly younger than non-responders (*M*s 17.04 and 17.25, respectively), *F*(1, 415) = 4.39, *p* = 0.037, η^2^ = 0.01 and were more likely to eat five portions of fruit and vegetables a day (*M*s 2.83 and 2.48, respectively), *F*(1, 414) = 8.40, *p* = 0.004, η^2^ = 0.02.

Chi-square test and one-way ANOVAs revealed no pre-intervention differences between participants across the four conditions in terms of gender, BMI, baseline frequency of fruit and vegetable consumption and negative urgency (all *p*s > 0.16). A significant difference between conditions was found for age *F*(1, 210) = 15.21, *p* < 0.001, η^2^ < 0.07, with participants in non-narrative conditions being slightly older (*M* = 17.31, SD = 0.98) than those in narrative conditions (*M* = 16.86 , SD = 0.73). A summary of descriptive statistics for the whole sample and for participants in the four study conditions is presented in Table 1. The frequencies of fruit and vegetable consumption by condition and bivariate correlations for study variables are included in the Supplemental Material.

**Table 1.** Summary of descriptive statistics by condition (*N* = 212).

Variable (range)	Whole sample, *M* (SD)	Non-narrative		Narrative		Summary of ANOVAs comparing intervention groups		
		Gain, *M* (SD)	Loss, *M* (SD)	Gain, *M* (SD)	Loss, *M* (SD)	*F*	η²	*p*
Baseline F&V (1–5)	2.85 (1.17)	3.05 (1.17)	2.62 (1.09)	2.96 (1.13)	2.79 (1.23)	1.21	0.02	0.31
Negative urgency (1–4)	2.71 (0.56)	2.72 (0.56)	2.73 (0.58)	2.58 (0.53)	2.79 (0.54)	1.71	0.02	0.16
Age (16–20)	17.04 (0.85)	17.34 (0.81)	17.27 (1.10)	16.86 (0.63)	16.85 (0.77)	5.14	0.07	0.002
BMI (15.20–38.20)	22.16 (3.95)	21.53 (3.59)	22.71 (4.54)	21.86 (3.77)	22.45 (3.92)	0.89	0.01	0.44
	Whole sample, *n* (%)	Non-narrative		Narrative		Summary of chi-square analyses for conditions: frame and format		
		Gain, *n* (%)	Loss, *n* (%)	Gain, *n* (%)	Loss, *n* (%)			
Gender	Male, 57 (27)	Male, 13 (32)	Male, 10 (22)	Male, 20 (35)	Male, 14 (20)	Frame, χ^2^(1) = 3.52, *p* = 0.06		
	Female, 155 (73)	Female, 30 (68)	Female, 34 (78)	Female, 37 (65)	Female, 54 (80)	Format, χ^2^(1) = 0.01, *p* = 0.97		

Baseline F&V refers to baseline frequency of fruit and vegetable consumption.

### Predicting fruit and vegetable consumption

The results of the regression analysis are displayed in Table 2. The predictors as a whole explained 33% of variance in the dependent variable. One control variable emerged as a significant predictor of fruit and vegetable consumption at time 2. Those consuming more fruit and vegetables in the past reported higher levels of fruit and vegetable consumption at follow-up (β = 0.53, *t* = 8.91, *p* < 0.001, 95% CI 0.41, 0.64). There were no significant associations between BMI, age and fruit and vegetable consumption at follow-up. Neither message format, message frame, nor negative urgency independently predicted fruit and vegetable consumption at follow-up. Results of regression analysis showed a significant three-way interaction between format, frame, and negative urgency, however (β = −0.24, *t* = −1.98, *p* = 0.05, 95% CI −0.95, −0.001), Δ*R*² = 0.013, Δ*F*(1, 201) = 3.90, *p* = 0.050.

**Table 2.** Moderated regression analysis predicting fruit and vegetable consumption at time 2 (*N* = 212).

Step	Variables entered	Step 1		Step 2		Step 3		Step 4	
		β	*T*	β	*t*	β	*t*	β	*t*
1	Baseline fruit and vegetable	0.53	8.91***	0.53	8.78***	0.51	8.63***	0.53	8.88***
	BMI	0.03	0.48	0.03	0.45	0.03	0.50	0.03	0.50
	Age	0.00	0.01	0.01	0.11	0.02	0.30	0.01	0.23
2	Negative urgency			−0.02	−0.37	0.24	2.23*	0.11	0.90
	Format			0.03	0.45	0.05	0.56	0.04	0.43
	Frame			−0.02	−0.28	−0.01	−0.12	−0.02	−0.25
3	Negative urgency × format					−0.27	−2.93**	−0.10	−0.83
	Negative urgency × frame					−0.09	−1.08	0.10	0.78
	Format × frame					−0.04	0.33	−0.03	−0.31
4	Negative urgency × format × frame							−0.24	−1.98*
*R* ^2^		0.28***		0.28***		0.31***		0.33***	
∆*F*		26.72***		0.15		3.22*		3.90*	
∆*R*^2^		0.28***		0.00		0.03*		0.01*	

β refers to standardized beta coefficients.

* *p* < 0.05. **p < 0.01. ***p < 0.001.

**Figure 1.**
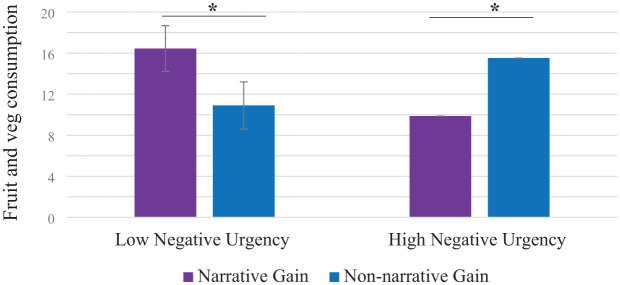
Two-way interaction between negative urgency and message format in the gain-frame condition on fruit and vegetable consumption (*N* = 212). Error bars show standard errors. *p < 0.05.

**Figure 2.**
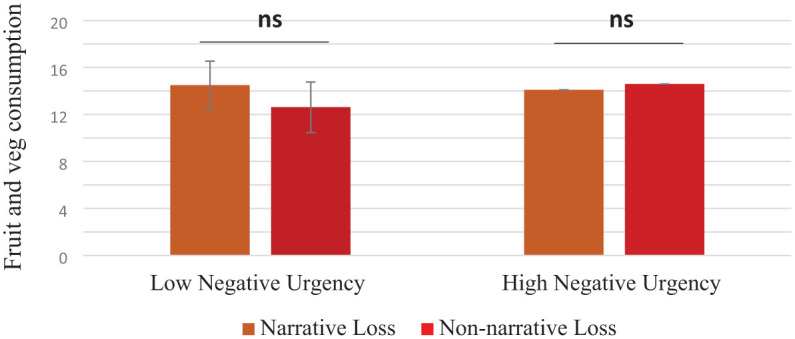
Two-way interaction between negative urgency and message format in the loss-frame condition on fruit and vegetable consumption (*N* = 212). Error bars show standard errors. ns: not significant.

Investigation of the three-way interaction using SPSS macro PROCESS (Hayes, 2013) showed that the format × negative urgency interaction was significant for participants in the gain-frame condition (β = −5.61, *t* = −3.45, *p* < 0.001, 95% CI −8.81, −2.40; see Figure 1), but not in the loss-frame condition (β = −1.24, *t* = 0.83, *p* = 0.41, 95% CI −4.19, 1.71; see Figure 2). Further probing of the format × negative urgency interaction in the gain-framed message condition revealed that for those with higher negative urgency, the non-narrative condition was associated with greater fruit and vegetables consumption compared to their counterparts with high levels of negative urgency in the narrative condition (β = −5.64, *t* = −2.39, *p* < 0.01, 95% CI −10.29, −0.99). Among participants at lower levels of negative urgency, those exposed to information in the narrative message condition reported greater intake of fruit and vegetables compared to those in the non-narrative message condition (β = 5.55, *t* = 2.49, *p* < 0.01, 95% CI 1.15, 9.96).

## Discussion

The aim of the current study was to conduct a preliminary examination of whether impulsivity, in the form of negative urgency, could impact the effects of message format and frame in persuading older adolescents to eat more fruit and vegetables. The results revealed a significant three-way interaction between negative urgency, message format and message frame. For older adolescents with higher levels of negative urgency, a gain-framed non-narrative message was associated with greater fruit and vegetable consumption than a gain-framed narrative. For older adolescents with lower levels of negative urgency, a gain-framed narrative message was associated with greater fruit and vegetable consumption compared to a gain-framed non-narrative message. For participants asked to read about the costs of a lack of fruit and vegetables in their diet (loss-frame), interaction effects were not found.

Our results provide initial support for our contention that high and low negative urgency adolescents likely have a usual way to process and therefore be persuaded by health messages. Their preference can manifest itself in the gain-frame, but not in the loss-frame, because, we argue, the latter produces a relatively strong emotional reaction that encourages recipients to adopt a particular way of processing that minimizes the effectiveness of emotional message content and forces effortful attention onto key message elements.

It appears that in the current study participants employed different message processing mechanisms when exposed to loss- and gain-framed information, yet both frames were effective in motivating their fruit and vegetable consumption. The frequency of fruit and vegetable consumption in the loss-frame was on a par with the average consumption in the gain-frame, with no main effect of frame being observed. It is evident that in the gain-framed condition low and high negative urgency adolescents were receptive more or less to health information depending on its format, whereas loss-framed information (narrative and non-narrative) potentially prompted all participants to ponder over health risks of insufficient fruit and vegetable consumption. Given that loss-framed (vs gain-framed) information has been shown to be more persuasive for those having pre-message intentions to increase fruit and vegetable intake (Godinho et al., 2016), it is possible that older adolescents in our study put an effort into processing of loss-framed messages because they have already considered eating more fruit and vegetables. However, we cannot make a firm conclusion about the mechanisms involved in the persuasion processes as we did not measure the type of message processing used by participants in the current study. Future research could help elucidate the nature of the processing used.

The findings of the present study contribute to a developing body of literature demonstrating that message recipient characteristics may determine the relative effectiveness of non-narrative and narrative health communications (Braverman, 2008; Slater and Rouner, 1996). This study showed that high negative urgency adolescents attending courses in post-compulsory education reported greater fruit and vegetable consumption after exposure to a gain-framed non-narrative (vs narrative). Potentially for older adolescents with high levels of negative urgency positively framed non-narratives represented less emotionally demanding format than narratives and thus were more persuasive. In contrast, gain-framed narratives (vs non-narratives) were more effective in motivating fruit and vegetable consumption among those low in negative urgency who tend to have good emotion regulation skills and can remain focused on a task. These results fit well with the notion that emotional and cognitive engagement are core elements of narrative persuasion, and narratives are influential for those who can connect with the story through emotions and mental imagery (Appel and Richter, 2010; Green and Brock, 2002; Mazzocco et al., 2010).

The current findings may be of interest to researchers and practitioners interested in optimizing the persuasive message design. Users of digital platforms, such as mobile apps and social media may be exposed to tailored educational information through personalized algorithms (i.e. algorithms that adapt content to individual user preferences and to responses to previously administered questionnaires (Mancone et al., 2024; Van der Heijden et al., 2024). Research has demonstrated that tailored information tends to be perceived as more personally relevant, is read with greater interest and processed more thoroughly than general intervention materials, which in turn may encourage dietary behaviour change (Briazu et al, 2024; Lustria et al., 2013). The results of the current explorative study can provide some guidance on how to tailor health information to adolescents, in order to maximize its persuasiveness. Specifically, after assessing adolescents’ levels of negative urgency, those reporting high levels of negative urgency could be exposed to health information in a gain-frame and in a non-narrative format, whereas those reporting low levels of negative urgency could be presented with gain-framed narrative messages. The current findings also showed that health information in the loss-frame was as persuasive as the gain-frame and neutralized the differences due to format found in the gain-frame. Therefore, if a ‘one size fits all’ communication campaign to promote fruit and vegetable consumption in adolescents was necessary, it could be more efficient to use only loss-framed messaging. However, the danger in doing so is that too strong or forceful a message may lead to resistance and lack of persuasion (Cho and Sands, 2011; LaVoie et al., 2017).

Our findings need to be considered in relation to certain limitations. In this study, we relied on the self-report measure of fruit and vegetable consumption over the 7 days post-intervention, which may be susceptible to demand characteristics and memory (Chan, 2009). Though self-reporting of dietary behaviour is sometimes problematic (Huang et al., 2005), this should not have occurred differentially across experimental conditions. Nevertheless, future research could usefully replicate the study using a different method of data collection (e.g. food diaries; see Brouwer and Mosack, 2015). Another limitation of this study is that we investigated the effects of loss- versus gain-framed non-narrative and narrative messages over a relatively short period of time (i.e. 7 days). Future research could investigate whether the pattern of results reported here would hold over a longer time frame. In the current study participants did not report their ethnicity and socioeconomic status. Future research could collect this information to investigate whether any message effects on fruit and vegetables consumption occur due to participants’ ethnic background or socioeconomic status.

In this study we found that as compared to those who dropped out, participants who remined in the study reported greater levels of baseline fruit and vegetable intake and were predominantly female (73.4%), which can constitute another limitation to the study. Past research has shown that adolescent girls show greater preferences for fruit and vegetables than for unhealthy foods (Wouters et al., 2010) and tend to eat a healthier diet than boys do (Blundell-Birtill and Hetherington, 2019). It is possible that among adolescents invited to take part in this study, girls were more interested in receiving information about the health outcomes of eating fruit and vegetables than boys were and thus, were more likely to take part in this study. Further, it is possible that potential interest in the research topic among female participants translated into the message effects on behaviour being observed regardless of its format. Future research with more equal gender distribution is warranted.

This study provides initial evidence that message format and frame may differently prompt fruit and vegetable consumption among older adolescents with lower and higher levels of negative urgency. As such the findings suggest that when developing dietary interventions for adolescents, researchers may wish to consider whether to match format and frame of educational information to adolescents’ levels of negative urgency as it may help to motivate behaviour change.

## Supplemental Material

sj-docx-1-hpq-10.1177_13591053251375237 – Supplemental material for Encouraging impulsive adolescents attending college to eat more fruit and vegetables: A preliminary investigation of negative urgency, message format and frame Supplemental material, sj-docx-1-hpq-10.1177_13591053251375237 for Encouraging impulsive adolescents attending college to eat more fruit and vegetables: A preliminary investigation of negative urgency, message format and frame by Joanna Slodkowska-Barabasz, Susan Churchill and Nik Chmiel in Journal of Health Psychology

sj-docx-2-hpq-10.1177_13591053251375237 – Supplemental material for Encouraging impulsive adolescents attending college to eat more fruit and vegetables: A preliminary investigation of negative urgency, message format and frame Supplemental material, sj-docx-2-hpq-10.1177_13591053251375237 for Encouraging impulsive adolescents attending college to eat more fruit and vegetables: A preliminary investigation of negative urgency, message format and frame by Joanna Slodkowska-Barabasz, Susan Churchill and Nik Chmiel in Journal of Health Psychology

sj-docx-3-hpq-10.1177_13591053251375237 – Supplemental material for Encouraging impulsive adolescents attending college to eat more fruit and vegetables: A preliminary investigation of negative urgency, message format and frame Supplemental material, sj-docx-3-hpq-10.1177_13591053251375237 for Encouraging impulsive adolescents attending college to eat more fruit and vegetables: A preliminary investigation of negative urgency, message format and frame by Joanna Slodkowska-Barabasz, Susan Churchill and Nik Chmiel in Journal of Health Psychology
