# Supplementary material for: Encouraging impulsive adolescents attending college to eat more fruit and vegetables: A preliminary investigation of negative urgency, message format and frame
Source: J Health Psychol. 2025 Oct 7;31(5):1855–70. doi: 10.1177/13591053251375237 (PMC13031376; doi:10.1177/13591053251375237)
Supplement: sj-docx-1-hpq-10.1177_13591053251375237 – Supplemental material for Encouraging impulsive adolescents attending college to eat more fruit and vegetables: A preliminary investigation of negative urgency, message format and frame [file sj-docx-1-hpq-10.1177_13591053251375237.docx]

Table 1. Persuasive Messages.

| Gain/Non-narrative | Please read the message carefully  Fruit and vegetable such as apples, bananas, kiwi fruits, melons, carrots, tomatoes and broccoli are part of a balanced diet and can help us stay healthy.  Government guidelines suggest we should eat 5 potions of fruit and vegetable a day – a portion can be an apple, 2 plums, one slice of melon, two broccoli spears, three heaped tablespoons of peas, or one medium tomato.  Research shows that young people aged 16-18 who eat fruit and vegetable, compared to those that do not, are at lower risk of many serious life-threatening diseases.  For example, research demonstrates that if you eat plenty of fruit and vegetable, you can decrease your risk of:   - Heart disease and stroke - High blood pressure - High cholesterol - Type 2 diabetes - Cancers (e.g., bowel cancer)   If you eat lots of fruit and vegetable - you can also gain potential health benefits, for example:   - Healthy looking skin and hair - Healthy weight   You may also and experience increased:   - Energy and vitality - Physical stamina - Concentration on mental tasks - Mood   Eat fruit and vegetable and feel great! |
| --- | --- |

Table 1. Continued.

| Gain/Narrative | Please read the message carefully  Sam is a 17 year old student at a sixth-form college in the South of England. Sam recently attended a lesson about the health benefits of eating fruit and vegetable.  “Dr Smith told us to be healthy we should eat 5 portions of fruit and veg a day. Dr Smith said a portion is an apple, 2 plums, one slice of melon, two broccoli spears, three heaped tablespoons of peas, or one medium tomato.  “Dr Smith also told us that young people who eat fruit and veg compared to those that do not, are at lower risk of many serious life-threatening diseases. Thinking back, I have been eating 5 portions a day – so I now realize that I am at decreased risk of heart disease and stroke, high blood pressure, high cholesterol, type 2 diabetes, and bowel cancer.  “It also seems that if I eat plenty of fruit and veg I can gain loads of health benefits such as healthy looking skin and hair, and healthy weight.  “Thinking now, I have enough fruit and vegetable in my diet, and I realize I have been feeling full of energy and vitality, and physical stamina. I have joined the local gym and attended spin classes which is great! I have been able to concentrate at school and actually I have been feeling very good recently.  “By eating fruit and veg I gain loads of health benefits.  Eat fruit and vegetable and feel great!” |
| --- | --- |

Table 1. Continued.

| Loss/Non-narrative | Please read the message carefully  Fruit and vegetable such as apples, bananas, kiwi fruits, melons, carrots, tomatoes and broccoli are part of a balanced diet and can help us stay healthy.  Government guidelines suggest we should eat 5 potions of fruit and vegetable a day – a portion can be an apple, 2 plums, one slice of melon, two broccoli spears, three heaped tablespoons of peas, or one medium tomato.  Research shows that young people aged 16-18 who do not eat fruit and vegetable, compared to those that do, are at higher risk of many serious life-threatening diseases.  For example, research demonstrates that if you do not eat plenty of fruit and vegetable, you can increase your risk of:   - Heart disease and stroke - High blood pressure - High cholesterol - Type 2 diabetes - Cancers (e.g., bowel cancer)   If you do not eat lots of fruit and vegetable – you can also lose potential health benefits, for example:   - Healthy looking skin and hair - Healthy weight   You may also and experience decreased:   - Energy and vitality - Physical stamina - Concentration on mental tasks - Mood   Don’t eat fruit and vegetable and feel rubbish! |
| --- | --- |

Table 1. Continued.

| Loss/Narrative | Please read the message carefully  Sam is a 17 year old student at a sixth-form college in the South of England. Sam recently attended a lesson about the costs of not eating fruit and vegetable.  “Dr Smith told us to be healthy we should eat 5 portions of fruit and veg a day. Dr Smith said a portion can be an apple, 2 plums, one slice of melon, two broccoli spears, three heaped tablespoons of peas, or one medium tomato.  “Dr Smith also told us that young people who do not eat fruit and veg compared to those that do, are at higher risk of many serious life-threatening diseases. Thinking back, I haven’t been eating 5 portions a day – so I now realize that I am at increased risk of heart disease and stroke, high blood pressure, high cholesterol, type 2 diabetes, and bowel cancer.  “It also seems that if I don’t eat plenty of fruit and veg I can lose loads of health benefits such as healthy looking skin and hair, and healthy weight.  “Thinking now, I don’t have enough fruit and vegetable in my diet, and I realize I have been feeling quite low in energy and vitality, and physical stamina. I haven’t bothered to go to the gym and gave up my spin classes. I haven’t been able to concentrate at school and I have been feeling pretty low recently.  “By not eating fruit and veg I risk lots of awful health issues.  Don’t eat fruit and vegetable and feel rubbish!” |
| --- | --- |
