# Supplementary material for: Encouraging impulsive adolescents attending college to eat more fruit and vegetables: A preliminary investigation of negative urgency, message format and frame
Source: J Health Psychol. 2025 Oct 7;31(5):1855–70. doi: 10.1177/13591053251375237 (PMC13031376; doi:10.1177/13591053251375237)
Supplement: sj-docx-2-hpq-10.1177_13591053251375237 – Supplemental material for Encouraging impulsive adolescents attending college to eat more fruit and vegetables: A preliminary investigation of negative urgency, message format and frame [file sj-docx-2-hpq-10.1177_13591053251375237.docx]

Table 3. Frequency of Fruit and Vegetable Consumption for High and Low Negative Urgency Adolescents in Study Conditions (*N* = 212).

| Level of Negative Urgency | Low Negative Urgency | | | | High Negative Urgency | | | |
| --- | --- | --- | --- | --- | --- | --- | --- | --- |
| Condition | Non-narrative Gain | Non-narrative Loss | Narrative Gain | Narrative Loss | Non-narrative gain | Non-narrative Loss | Narrative Gain | Narrative Loss |
| Frequency of F&V | 10.90 | 12.60 | 16.45 | 14.50 | 15.55 | 14.60 | 9.90 | 14.10 |

*Note.* Frequency of F&V refers to frequency of fruit and vegetable consumption measured at Time 2.
