# Supplementary material for: Encouraging impulsive adolescents attending college to eat more fruit and vegetables: A preliminary investigation of negative urgency, message format and frame
Source: J Health Psychol. 2025 Oct 7;31(5):1855–70. doi: 10.1177/13591053251375237 (PMC13031376; doi:10.1177/13591053251375237)
Supplement: sj-docx-3-hpq-10.1177_13591053251375237 – Supplemental material for Encouraging impulsive adolescents attending college to eat more fruit and vegetables: A preliminary investigation of negative urgency, message format and frame [file sj-docx-3-hpq-10.1177_13591053251375237.docx]

Table 4. Bivariate Correlations for Study Variables (*N* = 212).

|  | 1 | 2 | 3 | 4 | 5 |
| --- | --- | --- | --- | --- | --- |
| Baseline F&V | - |  |  |  |  |
| Time 2 F&V | .53** | - |  |  |  |
| Negative urgency | -.06 | -.04 | - |  |  |
| Age | .05 | .03 | -.08 | - |  |
| BMI | .03 | .05 | .05 | .11 | - |

*Note.* **p* < .05. ***p* < .01. F&V refers to fruit and vegetables.
